# Genetic association and meta-analysis of a schizophrenia GWAS variant rs10489202 in East Asian populations

**DOI:** 10.1038/s41398-018-0211-x

**Published:** 2018-08-07

**Authors:** Yongfeng Yang, Lu Wang, Lingyi Li, Wenqiang Li, Yan Zhang, Hong Chang, Xiao Xiao, Ming Li, Luxian Lv

**Affiliations:** 1 Department of Psychiatry, Henan Mental Hospital, The Second Affiliated Hospital of Xinxiang Medical University, Xinxiang, Henan China; 2 Henan Key Lab of Biological Psychiatry, Xinxiang Medical University, Xinxiang, Henan China; 3 Key Laboratory of Animal Models and Human Disease Mechanisms of the Chinese Academy of Sciences and Yunnan Province, Kunming Institute of Zoology, Kunming, Yunnan China; 4 Center for Excellence in Brain Science and Intelligence Technology, Chinese Academy of Sciences, Shanghai, China

## Abstract

Previous genome-wide association studies (GWAS) suggest that rs10489202 in the intron of *MPC2* (mitochondrial pyruvate carrier 2) is a risk locus for schizophrenia in Han Chinese populations. To validate this discovery, we conducted a replication analysis in an independent case-control sample of Han Chinese ancestry (437 cases and 2031 controls), followed by a meta-analytic investigation in multiple East Asian samples. In the replication analysis, rs10489202 showed marginal association with schizophrenia (two-tailed *P* = 0.071, OR = 1.192 for T allele); in the meta-analysis using a total of 14,340 cases and 20,349 controls from ten East Asian samples, rs10489202 was genome-wide significantly associated with schizophrenia (two-tailed *P* = 3.39 × 10^–10^, OR = 1.161 for T allele, under the fixed-effect model). We then performed an explorative investigation of the association between this SNP and bipolar disorder, as well as a major depressive disorder, and the schizophrenia-predisposing allele was associated with an increased risk of major depressive disorder in East Asians (two-tailed *P* = 2.49 × 10^–2^, OR = 1.103 for T allele). Furthermore, expression quantitative trait loci (eQTL) analysis in lymphoblastoid cell lines from East Asian donors (*N* = 85 subjects) revealed that rs10489202 was specifically and significantly associated with the expression of *TIPRL* gene (*P* = 5.67 × 10^–4^). Taken together, our data add further support for the genetic involvement of this genomic locus in the susceptibility to schizophrenia in East Asian populations, and also provide preliminary evidence for the underlying molecular mechanisms.

## Introduction

With ~0.70–1.10% prevalence worldwide and an estimated heritability of 80%^1,2^, schizophrenia has been recognized as an emerging public health issue. While its nature as a complex polygenic psychiatric disorder has hampered the efforts to understand its pathogenesis, studies analyzing the genetic architectures of schizophrenia in European populations have achieved great success^3^. However, the genetic components of schizophrenia in East Asians (e.g., Chinese and Japanese), the cohort occupying more than 20% of the world populations, remain less understood. Nevertheless, in 2011, two independent schizophrenia genome-wide association studies (GWAS) conducted in Han Chinese populations were published by Yue et al.^4^ and Shi et al.^5^, and they reported nine single-nucleotide polymorphisms (SNPs) showing genome-wide significant associations with this illness (see Table S1 for details). More recently, two schizophrenia GWAS conducted by Yu et al.^6^ and Li et al.^7^ in larger sample sets (partially overlapped with those from Yue et al.^4^ and Shi et al.^5^) have identified additional risk loci (see Table S2 for details).

The discovery of these schizophrenia risk SNPs in Han Chinese GWAS studies elicited numerous replication analyses. For example, we have previously conducted meta-analyses of the nine SNPs reported by Yue et al.^4^ and Shi et al.^5^ using four independent East Asian samples and observed nominal associations between a SNP rs10489202 in the second intron of *MPC2* (*MPC2*, containing seven exons and spanning 20.4 kb in the genome, encodes the mitochondrial pyruvate carrier 2 and locates in 1q24.2) and schizophrenia (two-tailed *P* = 0.0155)^8^, which was consistent with the original GWAS^5^. Although this SNP neither achieved genome-wide significance nor survived multiple corrections according to the number of SNPs tested (*N* = 9) in our replication meta-analysis^8^, it was likely reflecting the well-known “winner’s curse” effect hypothesis^9^ that the genetic effects of new association findings tend to be overestimated in the discovery study. More importantly, extra large samples are usually required to capture significant risk variants for polygenic disorders (e.g., schizophrenia)^10^, and the replication sample size was likely insufficient. As a result, more samples are necessary to validate the potential risk conferred by rs10489202 in schizophrenia. In the present study, we have conducted association analyses of three SNPs (rs10489202 and two additional SNPs rs9618 and rs203863) in the *MPC2* region in 437 schizophrenia cases and 2031 controls from Southern Han Chinese populations (these samples were not included in previous studies of the gene). Then, compared to our previous study^8^ of 3977 cases and 5589 controls, we performed an updated meta-analysis (14,340 cases and 20,349 controls) to confirm whether rs10489202 conferred the risk of schizophrenia in larger samples from East Asians. Given the well-known genetic overlap between schizophrenia and major mood disorders^11,12^, we have also examined the association of rs10489202 with major mood disorders in East Asians. At last, to understand the potential functional impact of the risk allele, we examined the expression quantitative trait loci (eQTL) associations in East Asian population from a published study^13^, and conducted a functional prediction analysis using the GWAVA (genome-wide annotation of variants) dataset^14^.

## Methods

### Association analysis in a new Chinese schizophrenia case-control sample

#### Subjects

In total, 437 schizophrenia cases and 2031 non-psychiatric controls were recruited from Southern China area. All subjects were of Han Chinese origin. The schizophrenia patients were diagnosed according to The Diagnostic and Statistical Manual of Mental Disorders, fourth edition (DSM-IV). Clinical information of each participant was obtained through reviewing his/her health records and interviewing family informants. Details regarding the onset and progression of clinical disorders, as well as schizophrenia-related symptoms were also collected. The patients having any history of alcohol or drug dependence, epilepsy, or other symptomatic psychoses were excluded. Unrelated non-psychiatric controls were recruited from the local communities and screened for any history of mental illnesses, drug abuse, or family history of psychiatric disorders. All individuals provided written informed consent for participation. The study was approved by the ethics committee of the Second Affiliated Hospital of Xinxiang Medical University.

#### SNP selection, SNP genotyping, and statistical analysis

For the association analysis in our case-control sample, a total of three SNPs were selected. SNP selection was based on previous studies^5,8^ and our preliminary analysis using data from the 1000-Human- Genomes dataset. We first selected rs10489202, a Han Chinese GWAS risk SNP, which was nominally significant in subsequent replications^5,8^. We then examined the linkage disequilibrium (LD) pattern of common SNPs (minor allele frequency higher than 0.05) in the *MPC2* region in East Asian populations from the 1000-Human-Genomes dataset, and based on the Tagger procedure implemented in Haploview (only pairwise tagging, *r*^*2*^ threshold = 0.8)^15^, we also selected two additional representative tagging SNPs (rs9618 and rs203863) for the following analysis based on the LD structure (Figure S1).

Genomic DNA of all participants was extracted from the venous blood using the standard phenol–chloroform method. The primers were designed to amplify the regions containing the selected SNPs. Genotyping of the SNPs was conducted using the SNaPShot method on an ABI 3130 automatic sequencer, as described elsewhere^16^. The genotypes of the tested SNPs were automatically called by GeneMarker V1.65 and manually verified. PCR amplification and genotyping were conducted again when the quality turned out to be low. The Haploview program (version 4.1) was applied to estimate the LD relationship between paired SNPs using the *r*^*2*^ algorithm, to determine the haplotype structures, and to test Hardy–Weinberg equilibrium (HWE) for each SNP^15^. Allelic associations were accessed with PLINK, and odds ratios (ORs) and 95% confidence intervals (95% CIs) corresponding to the effect allele were also calculated using PLINK^17^. All assays were performed blind to diagnosis and genotype.

### Meta-analysis of rs10489202 with schizophrenia

#### Eligibility criteria

In the current meta-analyses, studies were considered eligible when they met all three of the following criteria: (1) they must be case-control or family-based studies; (2) cases must be clinically diagnosed with schizophrenia and are free of comorbidity with other mental conditions; and (3) the samples in identified studies should be independent from each other.

#### Information sources

Statistical results (*P*-value, OR, and standard error (SE)) from two schizophrenia GWAS in Chinese populations were collected for this meta-analysis^5,18^. Candidate gene studies were also explored so that published data regarding the selected SNPs could be retrieved. Specifically, we carried out electronic searches used searching terms of “*MPC2*” or “rs10489202” in PubMed (1966 to present) and Web of Science (1899 to present). For the articles returned by the search, we excluded clearly irrelevant studies based on the information in the titles and abstracts. The remaining articles were then reviewed in their entireties for further eligibility evaluation. This search and exclusion process yielded three independent eligible samples from two studies^19,20^. Additionally, studies in Asian populations underwent a second-round examination in case that some studies have covered the SNPs of interest (including rs10489202) without showing “*MPC2*” or “rs10489202” in either titles or abstracts. This process identified three additional samples^21–23^ eligible for the meta-analysis. The last search was performed on December 12, 2017.

Eventually, besides the present case-control sample, a total of nine East Asian case-control datasets of rs10489202 from seven studies (Table 2) were utilized in the current meta-analysis, including samples from the original GWAS (3750 cases and 6468 controls) following replication (4383 cases and 4539 controls) by Shi et al.^5^, and data from independent studies by Ma et al.^23^ (975 cases and 1043 controls), Jin et al.^20^ (1093 cases and 1022 controls), Wong et al.^18^ (498 cases and 2025 controls), Saito et al.^21^ (1032 cases and 993 controls), Guan et al.^22^ (1471 cases and 1528 controls), and Li et al.^19^ (Zhuang Chinese samples with 300 cases and 300 controls, and Han Chinese samples with 400 cases and 400 controls). From these qualified samples, data of rs10489202 in 14,340 cases and 20,349 controls were extracted (also including the present case-control sample). All the patients were diagnosed with schizophrenia according to either ICD-10 or DSM-IV criteria, and control subjects were local volunteers with no records of mental illnesses. Descriptive information was extracted from each study, including (1) author(s) and year of publication; (2) methods (sample size and genotyping platform); (3) sample characteristics (i.e., sample area, definition of case status, mean age, and gender ratio); and (4) data of rs10489202.

#### Statistical analysis

In the meta-analysis, the pooled OR and 95% CIs were calculated based on the OR and SE of each individual sample. This calculation was conducted with RevMan software (v5.2) (http://tech.cochrane.org/revman/download) using the classical inverse-variance (IV) weighted methods, as described in our previous studies^24,25^. Heterogeneity between individual studies was estimated using the Cochran’s (Q) χ^2^ test by calculating the weighted sum of squares of deviations of individual OR estimates from the overall estimate^26^. In the event that *P*_Q_ < 0.10, statistically significant heterogeneity among ORs existed. Inconsistency across studies was quantified using the *I*^*2*^ metric (*I*^*2*^ = Q–d.f./Q), which represented the percentage of total variation across several studies due to heterogeneity^27^. *I*^*2*^ took values between 0 and 100%, and higher values denoted a greater degree of heterogeneity^27^.

To calculate the ORs and the corresponding 95% CIs in the combined samples, random-effect models are usually used in the presence of heterogeneity among individual studies (*P* < 0.10), and fixed-effect models are normally considered suitable when no heterogeneity is observed. A forest plot was generated to graphically present the pooled ORs and 95% CIs, with each square and the respective size representing one particular study and its weight.

To confirm that the current study had sufficient power for the proposed hypothesis, we performed power analysis using the Power and Sample Size Program^28^. The Begg’s funnel plot was drawn to identify any potential publication bias among the included studies using the “*metafor*” package^29^. In this plot, the X-axis displays the observed ORs, and the Y-axis shows the measure of precision (e.g., SE) of the observed ORs. In the absence of publication bias, the points should form a funnel shape, and the majority of the points should fall inside the pseudo-confidence region. To determine the effect of potential covariates (e.g., mean age and gender ratio) on the variation of ORs, we also conducted a regression analysis of ORs with these factors for individual studies.

### eQTL analysis

To investigate the potential impact of the schizophrenia risk SNP, we examined its association with the expression of nearby genes using eQTL databases. Currently, most available eQTL datasets used European or African-American samples^30,31^. Considering that many eQTLs are population-specific, we extracted eQTL data of East Asian individuals from a study by Stranger et al.^13^, in which genome-wide mRNA expression in lymphoblastoid cell lines of 726 individuals from eight global populations in the HapMap3 project was analyzed. To this end, eQTL results of 85 subjects of East Asian origin (Han Chinese and Japanese) were utilized in the current study. Descriptions of the participants and mRNA quantification processes can be found in the original report^13^.

## Results

### Association of MPC2 SNPs with schizophrenia in a new Chinese case-control sample

In our Han Chinese case-control sample, both rs10489202 and rs203863 in the *MPC2* region showed marginal associations with schizophrenia (rs10489202, two-tailed *P* = 0.071, OR = 1.192 for T allele; rs203863, two-tailed *P* = 0.077, OR = 1.172 for G-allele) (Table 1 presents the single SNP allelic analysis results of rs10489202, rs9618, and rs203863). Although no significant association was observed for the three SNPs in this sample, the direction of the allelic effect for rs10489202 was the same as those in previous studies^5,8^. Therefore, our data could be considered as a direct replication analysis of the previous studies. Notably, rs10489202 was nominally significant (one-tailed *P* = 0.036) in a one-tailed test. Overall, these results support the contention that rs10489202 is a potential risk SNP for schizophrenia in East Asian populations, but further studies are still required.

**Table 1:** Associations of the three SNPs in the *MPC2* region with schizophrenia in a Southern Chinese sample (437 cases and 2031 controls)

**CHR**	**SNP**	**POS**	**Alleles**	**Frequency**		***P*** **-value**	**OR**	**95% CIs**
				**Case**	**Control**			
1	rs9618	167893759	A/G	0.225	0.203	0.136	1.143	0.959–1.364
1	rs203863	167894681	G/A	0.228	0.201	0.0766	1.172	0.983–1.397
1	rs10489202	167903079	T/G	0.184	0.159	0.0708	1.192	0.985–1.442

*CHR* chromosome, *SNP* single-nucleotide polymorphism, *POS* position, *OR* odds ratio, *95% CIs* 95% confidence intervals

Note: The *P*-values are calculated based on a two-tailed test

The frequency is based on the first allele

### Meta-analysis of rs10489202 with schizophrenia in East Asian populations

#### Literature search and eligible studies

**Fig. 1:**
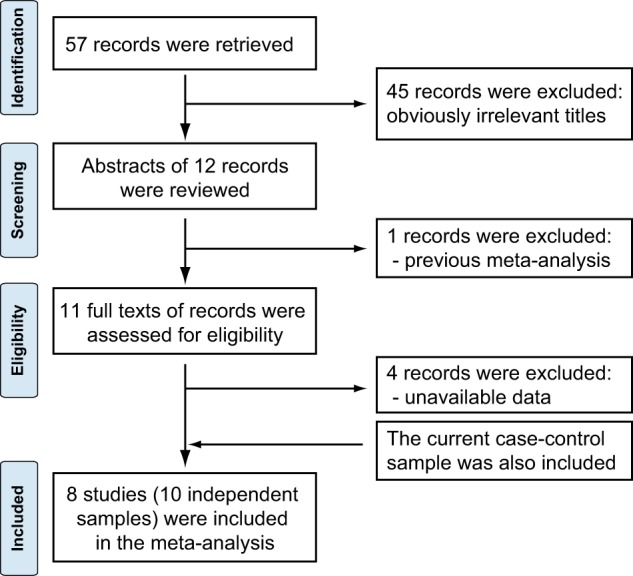
Literature search flowchart

The association of rs10489202 with schizophrenia in East Asian populations required further validation using larger samples; we therefore collected data from previous studies to perform a meta-analysis. Figure 1 presents a flowchart describing the literature search and selection process. The initial literature search returned 57 references, of which 45 publications with obviously irrelevant titles were excluded. The remaining 12 references underwent screening by abstracts, and one study was dropped out. For the 11 potential candidates, their full texts were then carefully examined, and seven studies including nine independent samples were finally considered eligible for the present meta-analysis (except the current case-control sample). The characteristics of the nine independent samples, including sample area, sample size, diagnostic criteria, genotyping method, and so on, are listed in Table 2. Overall, with the present case-control sample also included, we have collected data of 14,340 schizophrenia patients and 20,349 controls for the meta-analysis.

**Table 2:** Characteristics of samples on the association of rs10489202[T] with schizophrenia from the published studies

**Data source**	**Sample area**	**Diagnostic criteria**	**Schizophrenia cases**			**Healthy controls**			**Genotyping method**
			***N***	**Mean age**	**% Male**	***N***	**Mean age**	**% Male**	
Shi et al., 2011a^5^	China, multiple	ICD-10	3750	36.2 (12.4)	37.0	6468	60.9 (12.2)	57.0	Affymetrix GWAS 6.0
Shi et al., 2011b^5^	China, multiple	DSM-III-R	4383	35.6 (13.3)	56.6	4539	60.7 (11.2)	34.7	Ligation detection reaction (LDR)
Ma et al., 2012 ^23^	China, Hunan	DSM-IV	976	24.9 (8.3)	/	1043	37.4 (14.2)	/	SNaPShot
Jin et al.^20^	China, Jiangsu	DSM-IV	1093	47.9 (10.9)	63.8	1022	44.8 (10.2)	56.0	LDR–PCR
Wong et al.^18^	China, Hong Kong and Sichuan	DSM-IV	498	/	67.6	2025	/	33.3	Illumina Human610-Quad
Saito et al.^21^	Japan	DSM-IV	1032	46.8 (14.8)	51.7	993	49.7 (14.0)	48.9	Sequenom iPLEX Gold
Guan et al., 2015^22^	China, multiple	DSM-IV	1471	32.8 (9.0)	49.0	1528	33.4 (9.0)	46.7	Sequenom MassARRAY
Li et al., 2016a^19^	China, Guangxi	ICD-10	300	33.7 (12.0)	69.0	300	32.4 (12.3)	66.0	Sequenom MassARRAY
Li et al., 2016b^19^	China, Guangxi	ICD-10	400	32.3 (11.6)	67.2	400	33.1 (11.2)	62.0	Sequenom MassARRAY

#### Meta-analysis of the association between rs10489202 and schizophrenia

**Fig. 2:**
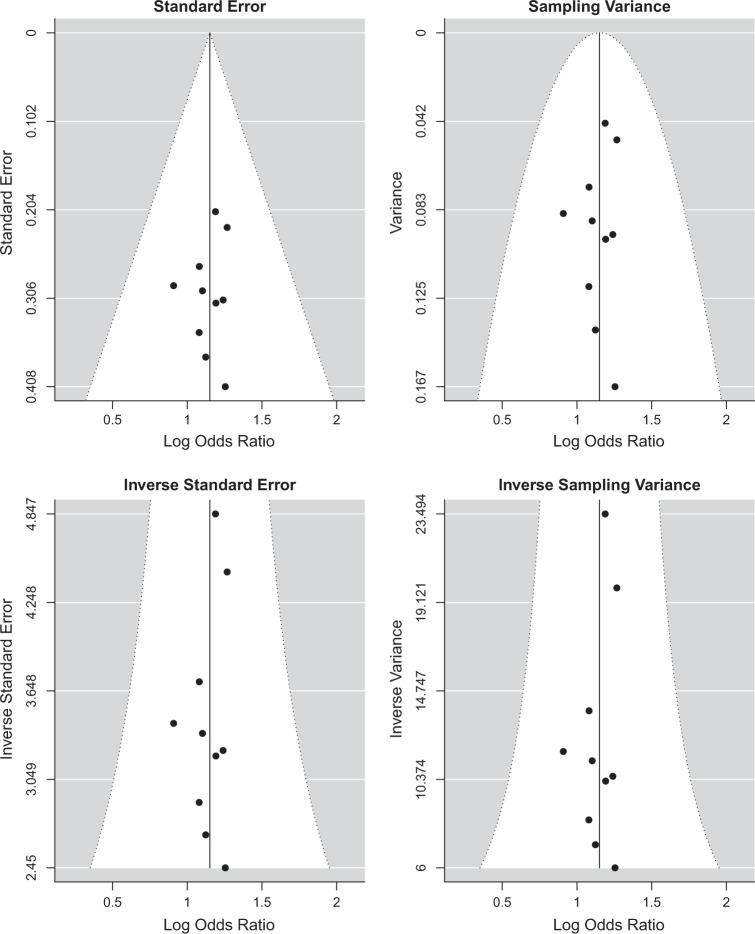
Begg’s funnel plot with pseudo 95% confidence limits for meta-analysis of rs10489202

The sample size of the present meta-analysis (14,340 cases and 20,349 controls) revealed > 90% power to detect a significant association for rs10489202, given its allele frequencies in East Asians and reported the effect size in the discovery of schizophrenia GWAS^5^. No evidence of significant publication bias was observed according to the Begg’s funnel plot (Fig. 2).

**Fig. 3:**
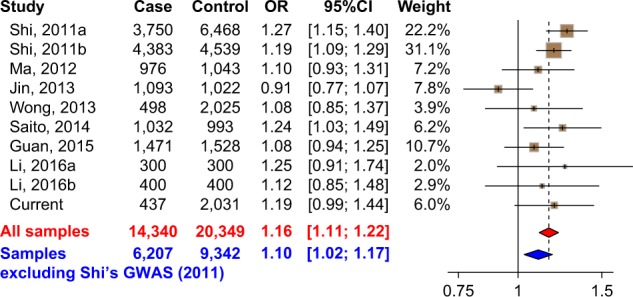
Forest plot of meta-analysis for rs10489202 with schizophrenia in East Asians

The complete results of the meta-analysis are shown in Fig. 3. Though rs10489202 was not associated with schizophrenia in any individual dataset besides those from Shi et al.^5^ and Saito et al.^21^ studies, we observed a significant association in the combination of all samples except for those from Shi et al.^5^ studies (this pooled sample is referred to as replication samples in the following discussion: two-tailed *P* = 0.00830, OR = 1.096 for T allele, in a total of 6207 cases and 9342 controls, Fig. 3). There was no significant heterogeneity between the studies (*P* = 0.331, *I*^*2*^ = 12.7%). This nominal significant result was likely due to the increased statistical power of the pooled sample compared with each individual sample, and the direction of allelic effects was the same as that from Shi et al. study^5^. When we combined all available samples together, in the fixed-effect model (test of heterogeneity, *P* = 0.119, *I*^*2*^ = 36.2%), the result achieved genome-wide significance (two-tailed *P* = 3.39 × 10^–10^, OR = 1.161 for T allele, in a total of 14,340 cases and 20,349 controls, Fig. 3). Notably, this *P*-value was even smaller compared with that in Shi’s discovery of GWAS (two-tailed *P* = 9.50 × 10^–9^). When we applied a more conservative random-effect model analysis, the level of statistical significance decreased (two-tailed *P* = 3.05 × 10^–5^, OR = 1.146 for T allele), but the observed OR of rs10489202 was still comparable to those of other schizophrenia risk variants discovered in large-scale meta-analyses^3^. Furthermore, we performed the “leave-one-out” sensitivity analysis to examine the contribution of each sample to the heterogeneity. We found that when the sample from Jin et al.^20^ was excluded from the overall meta-analysis, there was no heterogeneity among the remaining samples, and the meta-analytic result was the same between the random-effect model and the fixed-effect model (two-tailed *P* = 6.42 × 10^–12^, OR = 1.185 for T allele, in a total of 13,247 cases and 19,327 controls, Table S3). Therefore, the sample from Jin et al.^20^ was likely the source of heterogeneity in the meta-analysis. We also extracted the mean age and gender ratio of the participants in each replication sample, and performed regression analyses in SPSS 16.0 (SPSS Inc., Chicago, IL, USA) to test if these two factors (mean age and gender ratio) influenced the ORs of rs10489202. We found that neither factors (mean age: *P* > 0.30; gender ratio: *P* > 0.30) played significant roles in the risk association between rs10489202 and schizophrenia. Collectively, these data imply that rs10489202 is potentially an authentic schizophrenia-relevant locus in East Asian populations.

We also checked the published GWAS dataset (i.e., PGC2^3^) to examine whether rs10489202 was associated with schizophrenia in other populations such as the Europeans. However, the SNP was not associated with the illness in European individuals (33,640 cases and 43,456 controls, two-tailed *P* = 0.408, OR = 1.011 for T allele, Table 3)^3^. To explain the inconsistencies of these SNP associations among different ethnic populations, we first analyzed the distributions of rs10489202 in 53 populations worldwide using the HGDP Selection Browser (http://hgdp.uchicago.edu/)^32^. However, there were only slight differences of allelic frequencies between East Asians and Europeans (Figure S2). We then compared the LD patterns of the large genomic regions (~198.4 kb) spanning rs10489202 between East Asians and Europeans, and found that there were sharp differences in LD patterns between these two populations, which likely explained the failure of replications in Europeans (Figure S3). We have further confirmed this observation through examining the LD between rs10489202 and nearby SNPs in East Asian and European populations using the SNAP website (http://archive.broadinstitute.org/mpg/snap/ldplot.php) (Figure S4). We found that there were more SNPs in high LD (*r*^*2*^ > 0.9) with rs10489202 in Europeans, while in East Asians, SNPs in moderate (or higher) LD with rs10489202 (*r*^*2*^ > 0.5) spanned larger genomic regions. To test if there were other SNPs in this genomic region showing associations with schizophrenia in European populations, we retrieved an array of SNPs to obtain deeper screening resolution of the genomic area of interest from the PGC2 GWAS (33,640 cases and 43,456 controls)^3^. Overall, most of the SNPs in this region did not show significant association with schizophrenia (*P* > 0.05), but one SNP rs73033839 in low LD with rs10489202 (Europeans: *r*^*2*^ = 0.003, *D’* = 0.453) exhibited nominal association (*P* = 6.54 × 10^–5^, OR = 1.154, Figure S5). However, this SNP could not survive multiple correction on either genome-wide level or regional-wide level (i.e., there are 5138 tested SNPs in Figure S5, and the region-wide corrected *P*-value of rs73033839 is 0.336), and further analyses in larger samples are thus necessary. These data suggested that this genomic region might be an East Asian-specific risk region for schizophrenia. However, it is also possible that this genomic region contains an undiscovered rare causative variant, which may exhibit linkage with rs10489202 in East Asians and results in the significant associations observed; while in Europeans, the LD was distinct and thus no association was seen. Either hypothesis remains to be tested via deep-sequencing analysis and functional assays.

**Table 3:** Associations of rs10489202[T] with major psychiatric disorders in world populations from published studies

**References**	**Disease**	**Ethnicity**	**Cases**	**Controls**	***P*** **-value**	**OR**	**95% CIs**
3	Schizophrenia	European	33,640	43,456	0.408	1.011	0.986–1.036
21	BPD	East Asian	1012	993	0.901	1.010	0.830–1.230
35	BPD	European	7481	9250	0.782	0.992	0.941–1.047
34	MDD	East Asian	5303	5337	0.0249	1.103	1.024–1.188
36	MDD	European	9240	9519	0.485	0.983	0.936–1.032

*BPD* bipolar disorder, *MDD* major depressive disorder, *OR* odds ratio, *95% CIs* 95% confidence intervals

#### Association of rs10489202 with major mood disorders

Considering the well-known genetic overlap between major mood disorders (bipolar disorder and major depressive disorder) and schizophrenia^33^, we also explored the associations between rs10489202 and major mood disorders in East Asian populations using the published data. In fact, this SNP has been investigated for its role in bipolar disorder in a Japanese sample (1012 cases and 993 controls), but the authors did not obtain any evidence of association (two-tailed *P* = 0.901, OR = 1.01 for T allele, Table 3)^21^. However, after examination of the data from a recent GWAS of a major depressive disorder in Han Chinese population, we revealed that rs10489202 was significantly associated with this illness (two-tailed *P* = 2.49 × 10^–2^, OR = 1.103 for T allele, in a total of 5303 cases and 5337 controls, Table 3)^34^, and the risk allele was the same as that found in schizophrenia studies. Therefore, the association between this SNP and major mood disorders in East Asians is worth further investigative efforts. Interestingly, rs10489202 was not associated with bipolar disorder (two-tailed *P* = 0.782, OR = 0.992 for T allele, in a total of 7481 cases and 9250 controls, Table 3)^35^ or major depressive disorder (two-tailed *P* = 0.485, OR = 0.983 for T allele, in a total of 9240 cases and 9519 controls, Table 3)^36^ in European populations, corroborating the inter-ethnicity difference at this locus previously observed in schizophrenia studies. Nevertheless, this result adds further support for the shared genetic basis between schizophrenia and major depressive disorder in East Asian populations, while the association of this locus with bipolar disorder is less conclusive.

### Association of rs10489202 with gene expression and functional predictive analyses

The current study indicates that rs10489202 is likely involved in the risk of schizophrenia in East Asians, and dissecting the underlying biological mechanisms will provide further evidence for its role and the associated pathological mechanisms in this illness. Previous studies demonstrated that many SNPs confer the risk of schizophrenia via altering gene expression in the brain and blood tissues^3,37,38^, and thus we tested whether rs10489202 was an eQTL of specific gene(s).

**Fig. 4:**
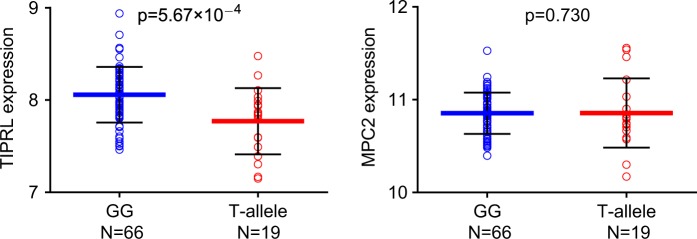
Association of rs10489202 with the expression of *TIPRL* and *MPC2* in Stranger et al. study^13^

After a thorough examination of currently available eQTL datasets, we retrieved the East Asian eQTL data from Stranger et al. study^13^ to examine the potential impact of rs10489202 on nearby gene expression. Although these data were obtained from lymphoblastoid cell lines, prior studies have proven their value in understanding the mechanisms of schizophrenia^39–42^. According to the data presented in Figure S6, there are 11 genes within 500 kb around rs10489202 and its LD SNPs. Given the low frequency of the rs10489202 minor allele [T] in East Asian populations, there are very few subjects carrying TT homozygotes in a moderate sample size (i.e., *N* = 3 in this eQTL sample), and we therefore combined all the T-allele carriers (including GT and TT groups) together (named T allele) for the analyses. Interestingly, in the HapMap3 East Asian samples (*N* = 85) from Stranger et al.^13^, rs10489202 was specifically and significantly associated with the expression of *TIPRL* gene (the encoded TOR-signaling pathway regulator, located in 1q24.2 and contains eight exons, spans 23.3 kb in the genome) in this genomic region (*P* = 5.67 × 10^–4^, Table S4 and Fig. 4a), with the risk of T allele predicting lower mRNA levels of *TIPRL*. rs10489202 was not associated with *MPC2* expression in this eQTL dataset (*P* = 0.730, Fig. 4b), which was not unexpected as one SNP is not necessarily regulating its nearest gene. It should be noted that *TIPRL* gene is located far from rs10489202 and its LD SNPs (at least the gene is outside of the high LD region with rs10489202, Figure S6). Since emerging evidence supports the hypothesis that SNPs in enhancer regions could regulate the expression of distal genes in the genome^43–45^, whether this eQTL association is a result of enhancer effect remains to be elucidated.

Although eQTL analysis usually provides essential information regarding the functional mechanisms underlying genomic loci, it is not sufficient to conclude that the statistically most significant SNP(s) in the risk genomic region is the causative variant(s) in a risk genomic region, given the high degree of LD between SNPs (i.e., rs10489202 in this study)^46^. For this reason, we explored the LD between rs10489202 and the surrounding SNPs in East Asian populations using the SNAP website with the East Asian panel from the 1000-Human-Genomes (pilot 1) dataset. Twenty SNPs (listed in Figure S6 and Table S5) were found to be in relatively high LD (*r*^*2*^ > 0.80) with rs10489202. Along with the idea that most schizophrenia risk SNPs exert their functions probably through affecting gene expression regulation^3^, we searched for potential functional SNPs through bioinformatic predictive analyses via synthesizing annotation information of *cis* and *trans* elements (e.g., DNase, H3K4me1, H3K4me3, and transcription factor-binding affinities) and genomic properties regarding GC-content, evolutionary conservation, and so on using the GWAVA dataset (http://www.sanger.ac.uk/sanger/StatGen_Gwava). This functional prediction showed that at least 4 out of the 21 candidate SNPs were located in the potential functional regions (Table S5), under the criteria that they contained transcription factor-binding sequences, as demonstrated by the ChIP-Seq method, located in the DNase hypersensitive sites or the histone modification regions, or being in exonic regions. Pinpointing the causative variant(s) and elucidating the mechanisms by which they affect gene expression should be explored in the future.

## Discussion

The improvement of genotyping technology and statistical methods has greatly enriched our knowledge of the genetic architectures of schizophrenia, but two principal problems in this field still require urgent attention. First, the most common risk variants show relatively small effect sizes in the genetic risk of schizophrenia, adding difficulties in understanding the illness; second, the poor replication of risk loci across different samples (sometimes even in the same genetic background) needs to be explained. Meta-analysis of statistics (OR and SE) of the original genotype data^47–50^ has been recognized as a feasible approach to obtain pooled large samples for additional information of the genetic basis of complex disorders. To this end, we have performed an independent replication analysis followed by the meta-analytic approach in the present study, and confirmed the association of a previous GWAS SNP rs10489202 with schizophrenia in East Asian populations. We also examined the functional outcome of the risk allele at rs10489202 using the eQTL data of lymphoblastoid cell lines, and found that it was significantly associated with the expression of *TIPRL*, implicating a potential schizophrenia susceptibility gene linked to this genetic locus. While this preliminary attempt indicates the involvement of *TIPRL* in the pathogenesis of schizophrenia, the exact function of this gene and its protein product in the human brain remains poorly reported. Being an inhibitory regulator of protein phosphatase-2A (PP2A), PP4, and PP6, *TIPRL* has been implicated in cancer^51^. Further studies analyzing its role in schizophrenia etiology are of great interest.

Although the *MPC2* gene is not related to the tested SNPs in the current eQTL analyses, our data do not deny the possibility that this gene and even other genes in the genomic region could be involved in schizophrenia. The *MPC2* gene encodes the mitochondrial pyruvate carrier 2. This protein is a component of the mitochondrial target of thiazolidinedione, and is known to regulate pyruvate entry into the mitochondria^52^. Its central role in cell metabolism thus makes it a viable target for insulin-related medications. Previous studies have reported an intercorrelation between insulin resistance and olanzapine treatment for schizophrenia^53^, warranting a potential significant impact of *MPC2* studies on the understanding and clinical management of schizophrenia.

While this study provides powerful evidence for the involvement of rs10489202 in schizophrenia susceptibility, certain limitations should also be acknowledged. First, a stratified analysis in specific psychiatric phenotypes is lacking due to the missing of detailed clinical information for each of the subjects included in this meta-analysis. While the present samples included different subtypes of schizophrenia, and such combinations could greatly increase the sample size and statistical power, the limitation of this approach could also camouflage the associations between SNPs and specific subtypes^54,55^. Second, the risk SNP rs10489202 was not associated with schizophrenia in European populations^3^, suggesting that there are potential genetic heterogeneities on this locus between different ethnic backgrounds. Thus, before we conclude that rs10489202 is a common schizophrenia risk variant in general world populations, further analyses revealing the genetic mechanism of this genomic locus in the risk of schizophrenia in Europeans are necessary^56^. Third, we also tested a limited number of SNPs in this genomic region, and it is unlikely that rs10489202 is the functional variant; whether there are underlying causative variants that explain the overall risk of associations in East Asians is still unclear. For example, rare mutations in specific genes have previously been highlighted in schizophrenia^57,58^, and such types of variants could only been identified through deep sequencing, which should be proposed in subsequent studies. Fourth, while the individuals included in our meta-analysis were recruited from different areas (e.g., provinces) and the samples from different studies were supposed to be independent, we were not able to thoroughly check detailed information of each participant, and could not exclude the possibility that certain individuals were recruited and used in more than one study. Therefore, extra caution is needed in interpreting the results. Fifth, we are also cautious about the interpretation of eQTL results in the present study. Although we identified a gene showing an association in lymphoblastoid cell lines, the lack of replication in independent samples is still a limitation; more importantly, since schizophrenia is a mental disorder that is supposed to originate from dysfunction of the brain, eQTL analysis in brain tissues is urgently needed. Finally, although rs10489202 showed genome-wide significant associations in the present study (14,340 cases and 20,349 controls), the SNP was not highlighted in the recent Han Chinese schizophrenia GWAS conducted by Li et al. (12,083 cases and 24,097 controls)^7^. However, a nearby proxy SNP rs6427113 (*r*^*2*^ = 0.345, *D’* = 0.614 with rs10489202 in East Asians) showed some degree of association in Li et al. study (*P* = 3.73 × 10^–5^, OR = 1.112), reflecting the previously reported genetic divergence between geographically distant populations in China^59^.

Collectively, our data add further support for the potential involvement of rs10489202 in the genetic risk of schizophrenia in Asian populations, and also provide suggestive evidence for its association with major depressive disorder in Han Chinese population. Further studies replicating this association in larger samples, as well as elucidating the underlying molecular mechanisms are necessary.

## Electronic supplementary material

Supplementary Tables

Supplementary Figures
