# Supplementary material for: Genetic association and meta-analysis of a schizophrenia GWAS variant rs10489202 in East Asian populations
Source: Transl Psychiatry. 2018 Aug 7;8:144. doi: 10.1038/s41398-018-0211-x (PMC6081446; doi:10.1038/s41398-018-0211-x)
Supplement: Supplementary file 1 — Supplementary Tables [file 41398_2018_211_MOESM1_ESM.pdf]

Table S1. Genome-wide significant SNPs in the GWASs reported by Yue et al.[1] and Shi et al.[2]

CHR	SNP	Allele	Frequency	Case	Control	P-value	OR	Ref.
6	rs1233710	T/C	0.350	4,773	7,207	4.76×10^{-11}	0.79	1
6	rs1635	A/C	0.350	4,773	7,207	6.91×10^{-12}	0.78	1
6	rs2142731	G/A	0.242	4,773	7,207	5.14×10^{-10}	0.79	1
11	rs11038167	A/C	0.375	4,773	7,207	1.09×10^{-11}	1.29	1
11	rs11038172	A/G	0.399	4,773	7,207	7.21×10^{-10}	1.25	1
11	rs835784	A/G	0.249	4,773	7,207	2.73×10^{-11}	1.27	1
1	rs10489202	T/G	0.131	8,133	11,007	9.50×10^{-09}	1.23	2
8	rs16887244	G/A	0.326	8,133	11,007	1.27×10^{-10}	0.84	2
8	rs1488935	A/G	0.320	8,133	11,007	5.06×10^{-09}	0.85	2

The frequency is based on the first allele.
Abbreviation:
CHR, chromosome; SNP, single nucleotide polymorphism; OR, odds ratio; Ref., references.

Table S2. Genome-wide significant SNPs in the GWASs reported by Li et al.[3] and Yu et al.[4]

CHR	SNP	Allele	Frequency	Case	Control	P-value	OR	Ref.
2	rs1518395	A/G	0.308	12,083	24,097	3.78×10^{-13}	0.862	3
2	rs78681500	T/C	0.063	12,083	24,097	3.08×10^{-10}	0.751	3
3	rs2073499	A/G	0.312	12,083	24,097	2.61×10^{-08}	0.899	3
6	rs111782145	C/T	0.037	12,083	24,097	1.80×10^{-08}	0.813	3
6	rs7757969	C/T	0.269	12,083	24,097	4.82×10^{-08}	1.110	3
6	rs4479915	G/C	0.254	12,083	24,097	4.82×10^{-09}	0.876	3
7	rs11534004	G/A	0.334	12,083	24,097	1.71×10^{-08}	0.890	3
2	rs1051061	G/A	0.412	8,723	12,813	1.14×10^{-10}	1.17	4
6	rs115070292	G/A	0.136	8,723	12,813	4.96×10^{-10}	0.77	4
10	rs10883795	C/T	0.496	8,723	12,813	7.94×10^{-10}	0.87	4
10	rs10883765	C/T	0.449	8,723	12,813	3.06×10^{-09}	0.87	4

The frequency is based on the first allele.
Abbreviation:
CHR, chromosome; SNP, single nucleotide polymorphism; OR, odds ratio; Ref., references.

Table S3. "Leave-one-out'' sensitivity analysis for meta-analysis

Excluded study	Remaining sample size		Heterogeneity	Random effect model			Fixed effect model		
	Case	Control		OR	95%CIs	Meta *P* value	OR	95%CIs	Meta *P* value
Shi, 2011a	10,590	13,881	p=0.250, I^2=21.7%	1.124	1.053-1.198	4.00×10^{-4}	1.132	1.074-1.194	4.03×10^{-6}
Shi, 2011b	9,957	15,810	p=0.092, I^2=41.4%	1.133	1.048-1.226	1.81×10^{-3}	1.148	1.086-1.215	1.37×10^{-6}
Ma, 2012	13,364	19,306	p=0.089, I^2=41.7%	1.149	1.071-1.233	1.13×10^{-4}	1.165	1.111-1.223	4.97×10^{-10}
Jin, 2013	13,247	19,327	p=0.743, I^2=0	1.185	1.129-1.244	6.42×10^{-12}	1.185	1.129-1.244	6.42×10^{-12}
Wong, 2013	13,842	18,324	p=0.089, I^2=41.7%	1.149	1.073-1.231	7.19×10^{-5}	1.164	1.110-1.221	3.39×10^{-10}
Saito, 2014	13,308	19,356	p=0.093, I^2=41.1%	1.136	1.060-1.218	3.31×10^{-4}	1.156	1.102-1.213	3.64×10^{-9}
Guan, 2015	12,869	18,821	p=0.111, I^2=38.6%	1.154	1.076-1.238	6.33×10^{-5}	1.171	1.115-1.230	3.39×10^{-10}
Li, 2016a	14,040	20,049	p=0.085, I^2=42.4%	1.141	1.066-1.221	1.33×10^{-4}	1.159	1.106-1.215	7.75×10^{-10}
Li, 2016b	13,940	19,949	p=0.081, I^2=43.0%	1.146	1.070-1.227	1.04×10^{-4}	1.162	1.108-1.218	4.66×10^{-10}
Current	13,903	18,318	p=0.081, I^2=43.0%	1.140	1.062-1.223	2.83×10^{-4}	1.159	1.105-1.216	1.74×10^{-9}

Table S4. Association of rs10489202 with nearby gene expression in the lymphoblastoid cell lines from 85 East Asian individuals[5]

	Beta estimate	Standard error	T-value	P-value
CD247	-0.00302	0.0361	-0.0837	0.934
CREG1	-0.0306	0.0730	-0.419	0.676
RCSD1	0.0232	0.0803	0.289	0.774
MPZL1	0.00714	0.0541	0.132	0.895
ADCY10	0.000448	0.0158	0.0283	0.977
MPC2	-0.0232	0.0671	-0.346	0.730
DCAF6	0.0505	0.0632	0.797	0.427
GPR161	-0.0278	0.0192	-1.443	0.153
TIPRL	-0.313	0.0868	-3.605	0.000567
SFT2D2	-0.00534	0.0264	-0.202	0.840
TBX19	0.0486	0.0278	1.747	0.0848

References

1. Yue WH, Wang HF, Sun LD, Tang FL, Liu ZH, Zhang HX *et al.* Genome-wide association study identifies a susceptibility locus for schizophrenia in Han Chinese at 11p11.2. *Nat Genet* 2011; **43**(12)**:** 1228-31.
2. Shi Y, Li Z, Xu Q, Wang T, Li T, Shen J *et al.* Common variants on 8p12 and 1q24.2 confer risk of schizophrenia. *Nat Genet* 2011; **43**(12)**:** 1224-7.
3. Li Z, Chen J, Yu H, He L, Xu Y, Zhang D *et al.* Genome-wide association analysis identifies 30 new susceptibility loci for schizophrenia. *Nat Genet* 2017; **49**(11)**:** 1576-83.
4. Yu H, Yan H, Li J, Li Z, Zhang X, Ma Y *et al.* Common variants on 2p16.1, 6p22.1 and 10q24.32 are associated with schizophrenia in Han Chinese population. *Mol Psychiatry* 2017; **22**(7)**:** 954-60.
5. Stranger BE, Montgomery SB, Dimas AS, Parts L, Stegle O, Ingle CE *et al.* Patterns of cis regulatory variation in diverse human populations. *PLoS Genet* 2012; **8**(4)**:** e1002639.

Table S5. Functional prediction analysis of the SNPs in high LD with rs10489202 (r2>0.8) using the GWAVA dataset (http://www.sanger.ac.uk/sanger/StatGen_Gwava)

#	1	2	3	4	5	6	7	8	9	10	11	12	13	14	15	16	17	18	19	20	21
**ID**	rs2420	rs17556487	rs10489202	rs1846416	rs4656564	rs7535138	rs2272911	rs718885	rs149912	rs12404574	rs4657727	rs12727036	rs1060041	rs7538581	rs12408205	rs17557135	rs17557162	rs11587594	rs66950781	rs17485889	rs4657730
**Chromosome**	1	1	1	1	1	1	1	1	1	1	1	1	1	1	1	1	1	1	1	1	1
**Position**	167,886,502	167,889,094	167,903,079	167,905,411	167,928,992	167,931,462	167,936,302	167,954,659	167,957,803	167,961,235	167,965,241	167,972,147	167,973,976	167,979,347	167,982,332	167,985,888	167,985,950	167,989,180	168,018,779	168,018,840	168,072,738
**Region score**	0.59	0.35	0.45	0.57	0.29	0.3	0.32	0.29	0.3	0.46	0.32	0.16	0.44	0.43	0.33	0.41	0.33	0.38	0.36	0.49	0.15
**TSS score**	0.55	0.14	0.27	0.36	0.06	0.09	0.08	0.25	0.09	0.06	0.12	0.01	0.3	0.11	0.1	0.11	0.13	0.14	0.07	0.09	0.21
**Unmatched score**	0.62	0.19	0.6	0.89	0.05	0.04	0.14	0.2	0.1	0.08	0.09	0.08	0.5	0.04	0.22	0.17	0.13	0.35	0.03	0.1	0.22
**Average GERP**	0.01334	-0.16541	-0.02107	-0.8374	0.12297	0.10577	-0.41347	-0.09049	-0.25916	-0.46098	0.26551	-0.04055	2.05732	-0.09049	-0.05581	0.56409	0.03975	-0.48486	-0.01168	-0.10122	-1.28459
**Average DAF**	0.00026	0.00087	0.00026	0.00031	0.00111	0.00024	0.00124	0.00072	0.00182	0.00023	0.00119	0.00156	0.00032	0.00106	0.00028	0.00041	0.0004	0.00029	0.00049	0.00049	0.00087
**Average het**	0.00046	0.00039	0.00045	0.00055	0.00053	0.00041	0.00042	0.00127	0.00105	0.00037	0.00069	0.00221	0.00056	0.00051	0.00048	0.00068	0.00066	0.00052	0.00085	0.00084	0.00124
**GERP**	-0.423	-0.771	1.17	-8.04	0.185	0.311	1.76	-0.241	-2.5	-6.41	1.33	-1.77	2.24	-0.717	-0.877	-1.51	1.61	-4.7	-0.451	1.32	-0.884
**%GC**	43.6%	36.6%	32.7%	73.3%	26.7%	35.6%	20.8%	44.6%	31.7%	33.7%	24.8%	32.7%	47.5%	37.6%	25.7%	27.7%	20.8%	33.7%	54.5%	36.6%	50.5%
**TSS distance**	-3048	-5640	-1941	-100	-8735	-6265	-1425	-13238	-10094	-6662	-2656	4248	6077	-5777	-2792	762	824	4054	4459	4520	1408
**SS distance**	-1095	-120	-1889	58	-6872	-4402	340	-2071	954	655	2560	331	-55	5313	-2684	696	758	272	-1348	-1287	-974
**ACCEPTOR**	-	-	-	-	-	-	-	-	-	-	-	-	-	-	-	-	-	-	-	-	-
**ATF3**	-	-	-	-	-	-	-	-	-	-	-	-	-	-	-	-	-	-	-	-	-
**BATF**	-	-	-	-	-	-	-	-	-	-	-	-	-	-	-	-	-	-	-	-	-
**BCL11A**	-	-	-	-	-	-	-	-	-	-	-	-	-	-	-	-	-	-	-	-	-
**BCL3**	-	-	-	-	-	-	-	-	-	-	-	-	-	-	-	-	-	-	-	-	-
**BCLAF1**	-	-	-	-	-	-	-	-	-	-	-	-	-	-	-	-	-	-	-	-	-
**BDP1**	-	-	-	-	-	-	-	-	-	-	-	-	-	-	-	-	-	-	-	-	-
**BHLHE40**	-	-	-	-	-	-	-	-	-	-	-	-	-	-	-	-	-	-	-	-	-
**BRCA1**	-	-	-	-	-	-	-	-	-	-	-	-	-	-	-	-	-	-	-	-	-
**BRF1**	-	-	-	-	-	-	-	-	-	-	-	-	-	-	-	-	-	-	-	-	-
**BRF2**	-	-	-	-	-	-	-	-	-	-	-	-	-	-	-	-	-	-	-	-	-
**CCNT2**	-	-	-	1	-	-	-	-	-	-	-	-	-	-	-	-	-	-	-	-	-
**CDS**	-	-	-	-	-	-	-	-	-	-	-	-	1	-	-	-	-	-	-	-	-
**CEBPB**	-	-	-	2	-	-	-	-	-	-	-	-	-	-	-	-	-	-	-	-	-
**CHD2**	-	-	-	-	-	-	-	-	-	-	-	-	-	-	-	-	-	-	-	-	-
**CTBP2**	-	-	-	-	-	-	-	-	-	-	-	-	-	-	-	-	-	-	-	-	-
**CTCF**	-	-	-	-	-	-	-	-	-	-	-	-	-	-	-	-	-	-	-	-	-
**CTCFL**	-	-	-	-	-	-	-	-	-	-	-	-	-	-	-	-	-	-	-	-	-
**CTCF_REG**	-	-	-	-	-	-	-	-	-	-	-	-	-	-	-	-	-	-	-	-	-
**DNase**	6	-	-	116	-	-	-	2	-	-	-	-	3	-	-	-	-	2	-	-	1
**DONOR**	-	-	-	-	-	-	-	-	-	-	-	-	-	-	-	-	-	-	-	-	-
**E2F1**	-	-	-	3	-	-	-	-	-	-	-	-	-	-	-	-	-	-	-	-	-
**E2F4**	-	-	-	2	-	-	-	-	-	-	-	-	-	-	-	-	-	-	-	-	-
**E2F6**	-	-	-	2	-	-	-	-	-	-	-	-	-	-	-	-	-	-	-	-	-
**EBF1**	-	-	-	2	-	-	-	-	-	-	-	-	-	-	-	-	-	-	-	-	-
**EGR1**	-	-	-	-	-	-	-	-	-	-	-	-	-	-	-	-	-	-	-	-	-
**ELF1**	-	-	-	2	-	-	-	-	-	-	-	-	-	-	-	-	-	-	-	-	-
**ELK4**	-	-	-	1	-	-	-	-	-	-	-	-	-	-	-	-	-	-	-	-	-
**ENH**	1	-	-	-	-	-	-	1	-	-	-	-	-	-	-	-	-	-	-	-	-

Table S5. Functional prediction analysis of the SNPs in high LD with rs10489202 (r2>0.8) using the GWAVA dataset (http://www.sanger.ac.uk/sanger/StatGen_Gwava)

#	1	2	3	4	5	6	7	8	9	10	11	12	13	14	15	16	17	18	19	20	21
ID	rs2420	rs17556487	rs10489202	rs1846416	rs4656564	rs7535138	rs2272911	rs718885	rs149912	rs12404574	rs4657727	rs12727036	rs1060041	rs7538581	rs12408205	rs17557135	rs17557162	rs11587594	rs66950781	rs17485889	rs4657730
EP300	1	-	-	-	-	-	-	-	-	-	-	-	-	-	-	-	-	-	-	-	-
ERALPHAA	-	-	-	-	-	-	-	-	-	-	-	-	-	-	-	-	-	-	-	-	-
ESRRA	-	-	-	-	-	-	-	-	-	-	-	-	-	-	-	-	-	-	-	-	-
ETS1	-	-	-	-	-	-	-	-	-	-	-	-	-	-	-	-	-	-	-	-	-
EXON	1	-	-	-	-	-	-	-	-	-	-	-	1	-	-	-	-	1	-	-	-
Eralphaa	-	-	-	-	-	-	-	-	-	-	-	-	-	-	-	-	-	-	-	-	-
FAIRE	1	-	1	18	-	2	-	2	-	1	-	-	1	-	-	-	-	-	-	-	-
FAM48A	-	-	-	-	-	-	-	-	-	-	-	-	-	-	-	-	-	-	-	-	-
FOS	-	-	-	1	-	-	-	-	-	-	-	-	-	-	-	-	-	-	-	-	-
FOSL1	-	-	-	-	-	-	-	-	-	-	-	-	-	-	-	-	-	-	-	-	-
FOSL2	-	-	-	-	-	-	-	-	-	-	-	-	-	-	-	-	-	-	-	-	-
FOXA1	1	-	-	1	-	-	-	-	-	-	-	-	-	-	-	-	-	-	-	-	-
FOXA2	1	-	-	-	-	-	-	-	-	-	-	-	-	-	-	-	-	-	-	-	-
GABPA	-	-	-	2	-	-	-	-	-	-	-	-	-	-	-	-	-	-	-	-	-
GATA1	-	-	-	1	-	-	-	-	-	-	-	-	1	-	-	-	-	-	-	-	-
GATA2	-	-	-	1	-	-	-	-	-	-	-	-	-	-	-	-	-	-	-	-	-
GATA3	-	-	-	-	-	-	-	-	-	-	-	-	-	-	-	-	-	-	-	-	-
GTF2B	-	-	-	-	-	-	-	-	-	-	-	-	-	-	-	-	-	-	-	-	-
GTF2F1	-	-	-	1	-	-	-	-	-	-	-	-	-	-	-	-	-	-	-	-	-
GTF3C2	-	-	-	-	-	-	-	-	-	-	-	-	-	-	-	-	-	-	-	-	-
H2AFZ	-	-	-	6	-	-	-	1	-	-	-	-	-	-	-	-	-	-	-	-	1
H3K27ac	1	-	7	10	-	-	-	1	-	-	-	-	-	-	-	-	-	-	-	-	-
H3K27me3	-	-	-	-	-	-	-	-	-	-	-	-	-	-	-	-	-	-	-	-	1
H3K36me3	7	2	-	-	-	-	5	4	-	11	5	5	13	-	8	5	5	16	4	4	6
H3K4me1	-	-	6	-	-	-	-	3	-	-	1	-	-	-	-	-	-	-	-	-	1
H3K4me2	-	-	13	13	-	-	-	2	-	-	1	-	-	-	-	-	-	-	-	-	-
H3K4me3	-	-	10	13	-	-	-	1	-	-	-	-	-	-	-	-	-	-	-	-	-
H3K79me2	-	1	6	5	5	4	2	3	1	-	-	-	-	-	-	-	-	-	-	-	-
H3K9ac	-	1	9	13	-	-	-	1	-	-	-	-	-	-	-	-	-	-	-	-	1
H3K9me1	-	-	-	-	-	-	-	-	-	-	-	-	-	-	-	-	-	-	-	-	-
H3K9me3	-	-	-	-	-	-	-	-	-	-	-	-	-	-	-	-	-	-	-	-	-
H4K20me1	-	-	1	1	2	1	-	-	-	-	-	-	-	-	-	-	-	-	-	-	-
HDAC2	-	-	-	-	-	-	-	-	-	-	-	-	-	-	-	-	-	-	-	-	-
HDAC8	-	-	-	-	-	-	-	-	-	-	-	-	-	-	-	-	-	-	-	-	-
HEY1	-	-	-	-	-	-	-	-	-	-	-	-	-	-	-	-	-	-	-	-	-
HMGN3	-	-	-	1	-	-	-	-	-	-	-	-	-	-	-	-	-	-	-	-	-
HNF4A	-	-	-	-	-	-	-	-	-	-	-	-	-	-	-	-	-	-	-	-	-
HNF4G	1	-	-	-	-	-	-	-	-	-	-	-	-	-	-	-	-	-	-	-	-
HSF1	-	-	-	-	-	-	-	-	-	-	-	-	-	-	-	-	-	-	-	-	-
INTRON	-	1	1	1	1	1	1	1	1	1	1	1	-	1	1	1	1	1	1	1	1
IRF1	-	-	-	1	-	-	-	-	-	-	-	-	-	-	-	-	-	-	-	-	-

Table S5. Functional prediction analysis of the SNPs in high LD with rs10489202 (r2>0.8) using the GWAVA dataset (http://www.sanger.ac.uk/sanger/StatGen_Gwava)

#	1	2	3	4	5	6	7	8	9	10	11	12	13	14	15	16	17	18	19	20	21
ID	rs2420	rs17556487	rs10489202	rs1846416	rs4656564	rs7535138	rs2272911	rs718885	rs149912	rs12404574	rs4657727	rs12727036	rs1060041	rs7538581	rs12408205	rs17557135	rs17557162	rs11587594	rs66950781	rs17485889	rs4657730
IRF3	-	-	-	-	-	-	-	-	-	-	-	-	-	-	-	-	-	-	-	-	-
IRF4	-	-	-	-	-	-	-	-	-	-	-	-	-	-	-	-	-	-	-	-	-
JUN	-	-	-	-	-	-	-	-	-	-	-	-	-	-	-	-	-	-	-	-	-
JUNB	-	-	-	-	-	-	-	-	-	-	-	-	-	-	-	-	-	-	-	-	-
JUND	-	-	-	-	-	-	-	-	-	-	-	-	-	-	-	-	-	-	-	-	-
KAT2A	-	-	-	-	-	-	-	-	-	-	-	-	-	-	-	-	-	-	-	-	-
MAFF	-	-	-	-	-	-	-	-	-	-	-	-	-	-	-	-	-	-	-	-	-
MAFK	-	-	-	-	-	-	-	-	-	-	-	-	-	-	-	-	-	-	-	-	-
MAX	-	-	-	3	-	-	-	-	-	-	-	-	-	-	-	-	-	-	-	-	-
MEF2A	-	-	-	1	-	-	-	-	-	-	-	-	-	-	-	-	-	-	-	-	-
MEF2_complex	-	-	-	-	-	-	-	-	-	-	-	-	-	-	-	-	-	-	-	-	-
MXI1	-	-	-	2	-	-	-	-	-	-	-	-	-	-	-	-	-	-	-	-	-
MYC	-	-	-	2	-	-	-	-	-	-	-	-	-	-	-	-	-	-	-	-	-
NANOG	-	-	-	-	-	-	-	-	-	-	-	-	-	-	-	-	-	-	-	-	-
NFE2	-	-	-	-	-	-	-	-	-	-	-	-	-	-	-	-	-	-	-	-	-
NFKB1	-	-	-	1	-	-	-	-	-	-	-	-	-	-	-	-	-	-	-	-	-
NFYA	-	-	-	1	-	-	-	-	-	-	-	-	-	-	-	-	-	-	-	-	-
NFYB	-	-	-	1	-	-	-	-	-	-	-	-	-	-	-	-	-	-	-	-	-
NR2C2	-	-	-	-	-	-	-	-	-	-	-	-	-	-	-	-	-	-	-	-	-
NR3C1	-	-	-	-	-	-	-	-	-	-	-	-	-	-	-	-	-	-	-	-	-
NR4A1	-	-	-	-	-	-	-	-	-	-	-	-	-	-	-	-	-	-	-	-	-
NRF1	-	-	-	5	-	-	-	-	-	-	-	-	-	-	-	-	-	-	-	-	-
PAX5	-	-	-	1	-	-	-	-	-	-	-	-	-	-	-	-	-	-	-	-	-
PBX3	-	-	-	-	-	-	-	-	-	-	-	-	-	-	-	-	-	-	-	-	-
POLR2A	-	-	-	4	-	-	-	1	-	-	-	-	-	-	-	-	-	-	-	-	-
POLR2A_elongatin	-	-	-	-	-	-	-	-	-	-	-	-	-	-	-	-	-	-	-	-	-
POLR3A	-	-	-	-	-	-	-	-	-	-	-	-	-	-	-	-	-	-	-	-	-
POU2F2	-	-	-	-	-	-	-	-	-	-	-	-	-	-	-	-	-	-	-	-	-
POU5F1	-	-	-	-	-	-	-	-	-	-	-	-	-	-	-	-	-	-	-	-	-
PPARGC1A	-	-	-	-	-	-	-	-	-	-	-	-	-	-	-	-	-	-	-	-	-
PRDM1	-	-	-	1	-	-	-	-	-	-	-	-	-	-	-	-	-	-	-	-	-
RAD21	-	-	-	-	-	-	-	-	-	-	-	-	-	-	-	-	-	-	-	-	-
RDBP	-	-	-	-	-	-	-	-	-	-	-	-	-	-	-	-	-	-	-	-	-
REP	1	2	1	-	2	2	1	-	2	1	3	2	-	1	2	1	1	-	1	1	3
REST	-	-	-	-	-	-	-	-	-	-	-	-	-	-	-	-	-	-	-	-	-
RFX5	-	-	-	3	-	-	-	-	-	-	-	-	-	-	-	-	-	-	-	-	-
RXRA	1	-	-	-	-	-	-	-	-	-	-	-	-	-	-	-	-	-	-	-	-
SETDB1	-	-	-	-	-	-	-	-	-	-	-	-	-	-	-	-	-	-	-	-	-
SIN3A	-	-	-	-	-	-	-	-	-	-	-	-	-	-	-	-	-	-	-	-	-
SIRT6	-	-	-	-	-	-	-	-	-	-	-	-	-	-	-	-	-	-	-	-	-
SIX5	-	-	-	1	-	-	-	-	-	-	-	-	-	-	-	-	-	-	-	-	-

Table S5. Functional prediction analysis of the SNPs in high LD with rs10489202 (r2>0.8) using the GWAVA dataset (http://www.sanger.ac.uk/sanger/StatGen_Gwava)

#	1	2	3	4	5	6	7	8	9	10	11	12	13	14	15	16	17	18	19	20	21
ID	rs2420	rs17556487	rs10489202	rs1846416	rs4656564	rs7535138	rs2272911	rs718885	rs149912	rs12404574	rs4657727	rs12727036	rs1060041	rs7538581	rs12408205	rs17557135	rs17557162	rs11587594	rs66950781	rs17485889	rs4657730
SLC22A2	-	-	-	-	-	-	-	-	-	-	-	-	-	-	-	-	-	-	-	-	-
SMARCA4	-	-	-	-	-	-	-	-	-	-	-	-	-	-	-	-	-	-	-	-	-
SMARCB1	-	-	-	-	-	-	-	-	-	-	-	-	-	-	-	-	-	-	-	-	-
SMARCC1	-	-	-	-	-	-	-	-	-	-	-	-	-	-	-	-	-	-	-	-	-
SMARCC2	-	-	-	-	-	-	-	-	-	-	-	-	-	-	-	-	-	-	-	-	-
SMC3	-	-	-	-	-	-	-	-	-	-	-	-	-	-	-	-	-	-	-	-	-
SP1	1	-	-	3	-	-	-	-	-	-	-	-	-	-	-	-	-	-	-	-	-
SP2	-	-	-	-	-	-	-	-	-	-	-	-	-	-	-	-	-	-	-	-	-
SPI1	-	-	-	-	-	-	-	-	-	-	-	-	-	-	-	-	-	-	-	-	-
SREBF1	-	-	-	1	-	-	-	-	-	-	-	-	-	-	-	-	-	-	-	-	-
SREBF2	-	-	-	-	-	-	-	-	-	-	-	-	-	-	-	-	-	-	-	-	-
SRF	-	-	-	-	-	-	-	-	-	-	-	-	-	-	-	-	-	-	-	-	-
START	-	-	-	-	-	-	-	-	-	-	-	-	-	-	-	-	-	-	-	-	-
STAT1	-	-	-	-	-	-	-	-	-	-	-	-	-	-	-	-	-	-	-	-	-
STAT2	-	-	-	-	-	-	-	-	-	-	-	-	-	-	-	-	-	-	-	-	-
STAT3	-	-	-	1	-	-	-	-	-	-	-	-	-	-	-	-	-	-	-	-	-
STOP	-	-	-	-	-	-	-	-	-	-	-	-	-	-	-	-	-	-	-	-	-
SUZ12	-	-	-	-	-	-	-	-	-	-	-	-	-	-	-	-	-	-	-	-	-
TAF1	-	-	-	-	-	-	-	-	-	-	-	-	-	-	-	-	-	-	-	-	-
TAF7	-	-	-	-	-	-	-	-	-	-	-	-	-	-	-	-	-	-	-	-	-
TAL1	-	-	-	-	-	-	-	-	-	-	-	-	-	-	-	-	-	-	-	-	-
TBP	-	-	-	2	-	-	-	-	-	-	-	-	-	-	-	-	-	-	-	-	-
TCF12	-	-	-	1	-	-	-	-	-	-	-	-	-	-	-	-	-	-	-	-	-
TCF7L2	-	-	-	-	-	-	-	-	-	-	-	-	-	-	-	-	-	-	-	-	-
TFAP2A	-	-	-	1	-	-	-	-	-	-	-	-	-	-	-	-	-	-	-	-	-
TFAP2C	-	-	-	1	-	-	-	-	-	-	-	-	-	-	-	-	-	-	-	-	-
THAP1	-	-	-	2	-	-	-	-	-	-	-	-	-	-	-	-	-	-	-	-	-
TRAN	4	4	-	-	4	4	5	4	4	5	3	4	6	5	4	5	5	6	5	5	2
TRIM28	-	-	-	-	-	-	-	-	-	-	-	-	-	-	-	-	-	-	-	-	-
TSS	-	-	-	6	-	-	-	-	-	-	-	-	-	-	-	-	-	-	-	-	-
TSS_FLANK	-	-	2	-	-	-	-	-	-	-	-	-	-	-	-	-	-	-	-	-	-
USF1	-	-	-	1	-	-	-	-	-	-	-	-	-	-	-	-	-	-	-	-	-
USF2	-	-	-	-	-	-	-	-	-	-	-	-	-	-	-	-	-	-	-	-	-
UTR3	1	-	-	-	-	-	-	-	-	-	-	-	-	-	-	-	-	-	-	-	-
UTR5	-	-	-	-	-	-	-	-	-	-	-	-	-	-	-	-	-	-	-	-	-
WEAK_ENH	-	-	-	-	-	-	-	-	-	-	-	-	-	-	-	-	-	-	-	-	-
WRNIP1	-	-	-	-	-	-	-	-	-	-	-	-	-	-	-	-	-	-	-	-	-
XRCC4	-	-	-	-	-	-	-	-	-	-	-	-	-	-	-	-	-	-	-	-	-
YY1	-	-	-	9	-	-	-	-	-	-	-	-	-	-	-	-	-	-	-	-	-
ZBTB33	-	-	-	-	-	-	-	-	-	-	-	-	-	-	-	-	-	-	-	-	-
ZBTB7A	-	-	-	-	-	-	-	-	-	-	-	-	-	-	-	-	-	-	-	-	-

Table S5. Functional prediction analysis of the SNPs in high LD with rs10489202 (r2>0.8) using the GWAVA dataset (http://www.sanger.ac.uk/sanger/StatGen_Gwava)

#	1	2	3	4	5	6	7	8	9	10	11	12	13	14	15	16	17	18	19	20	21
ID	rs2420	rs17556487	rs10489202	rs1846416	rs4656564	rs7535138	rs2272911	rs718885	rs149912	rs12404574	rs4657727	rs12727036	rs1060041	rs7538581	rs12408205	rs17557135	rs17557162	rs11587594	rs66950781	rs17485889	rs4657730
ZEB1	-	-	-	1	-	-	-	-	-	-	-	-	-	-	-	-	-	-	-	-	-
ZNF143	-	-	-	-	-	-	-	-	-	-	-	-	-	-	-	-	-	-	-	-	-
ZNF263	-	-	-	-	-	-	-	-	-	-	-	-	-	-	-	-	-	-	-	-	-
ZNF274	-	-	-	-	-	-	-	-	-	-	-	-	-	-	-	-	-	-	-	-	-
ZZZ3	-	-	-	-	-	-	-	-	-	-	-	-	-	-	-	-	-	-	-	-	-
bound_motifs	-	-	-	3	-	-	-	-	-	-	-	-	-	-	-	-	-	-	-	-	-
cpg_island	-	-	-	1	-	-	-	-	-	-	-	-	-	-	-	-	-	-	-	-	-
dnase_fps	1	-	-	29	-	-	-	1	-	-	-	-	-	-	-	-	-	-	-	-	-
in_cpg	-	-	-	-	-	-	-	-	-	-	-	-	-	-	-	-	-	-	-	-	-
pwm	-	-	-	-	-	-	-	-	-	-	-	-	-	-	-	-	-	-	-	-	-
repeat	1	-	-	-	1	1	-	1	-	-	1	1	-	-	-	-	-	-	1	-	-
seq_A	1	-	-	-	-	-	-	1	1	1	-	1	-	1	1	1	-	1	-	-	1
seq_C	-	-	-	-	-	-	1	-	-	-	-	-	1	-	-	-	-	-	1	-	-
seq_G	-	-	1	1	-	-	-	-	-	-	-	-	-	-	-	-	-	-	-	1	-
seq_T	-	1	-	-	1	1	-	-	-	-	1	-	-	-	-	-	1	-	-	-	-
