# Supplementary material for: Genetic association and meta-analysis of a schizophrenia GWAS variant rs10489202 in East Asian populations
Source: Transl Psychiatry. 2018 Aug 7;8:144. doi: 10.1038/s41398-018-0211-x (PMC6081446; doi:10.1038/s41398-018-0211-x)

Figure S1. Haplotype block structure of the common SNPs in MPC2 in East Asian populations. The LD value was shown as r^2. The SNPs tested in this study were highlighted with a blue rectangle.

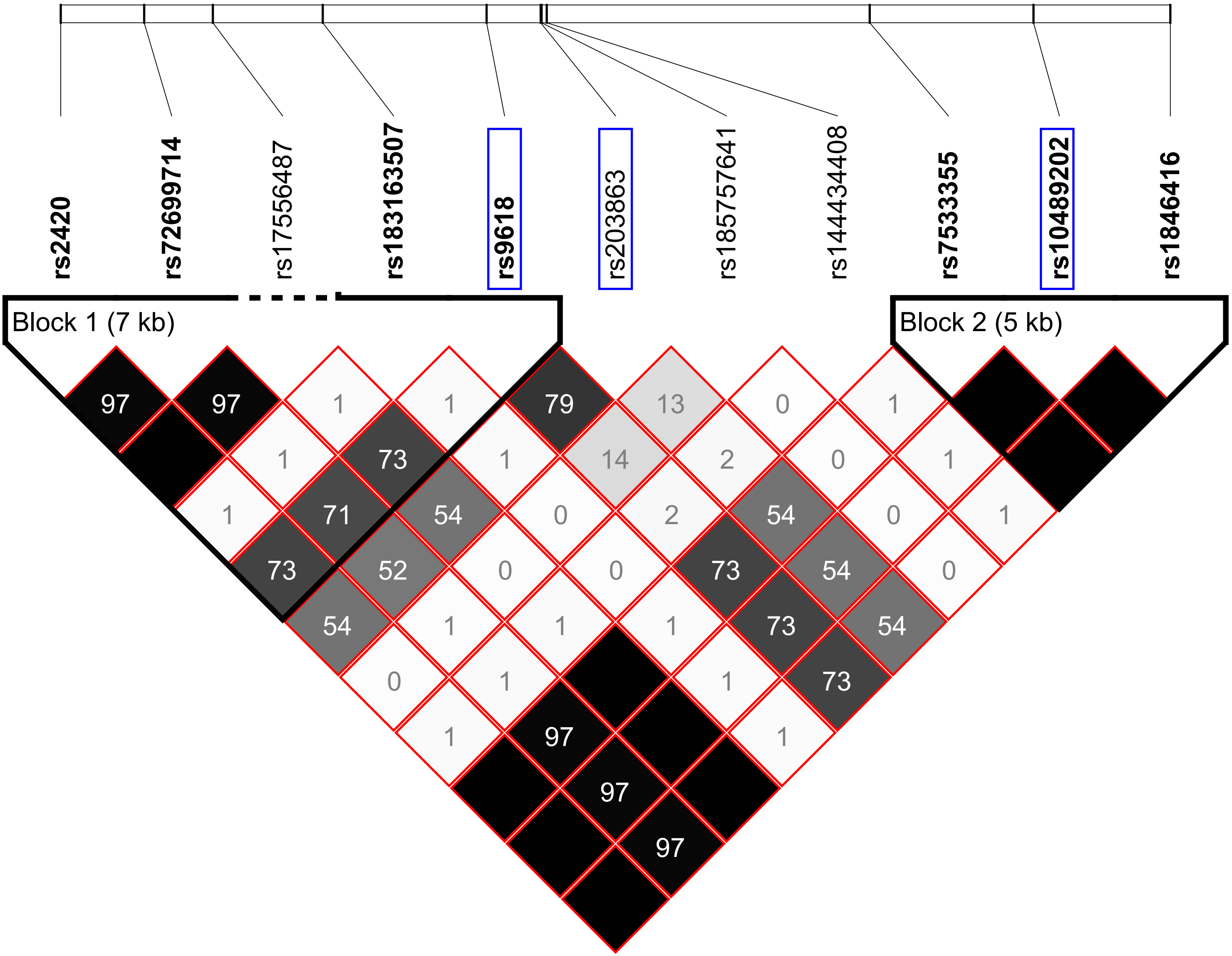

Figure S2. Global distributions of rs10489202 in 53 world populations

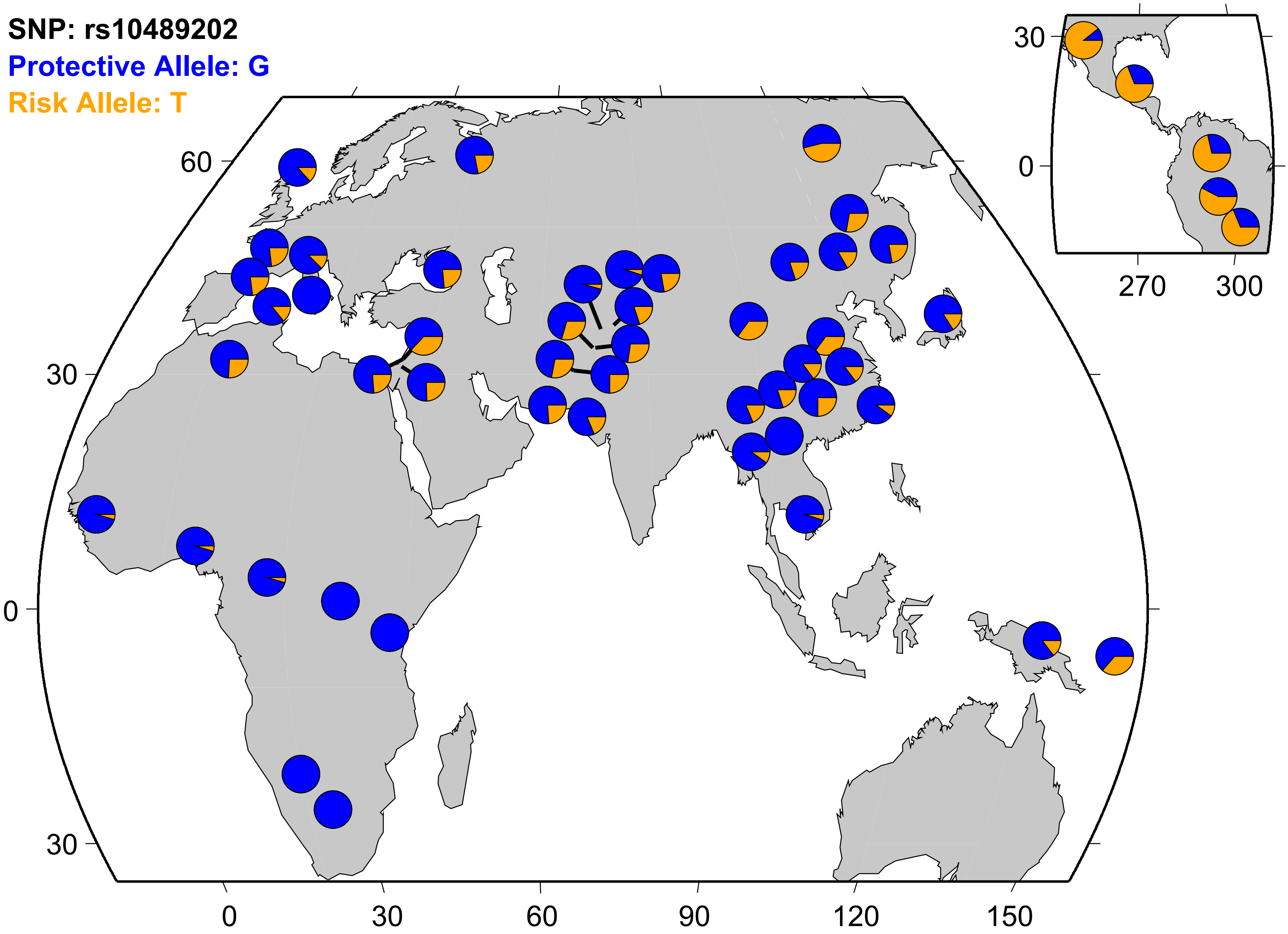

Figure S3. Comparisons of LD patterns of the genomic regions covering rs10489202 between East Asians and Europeans

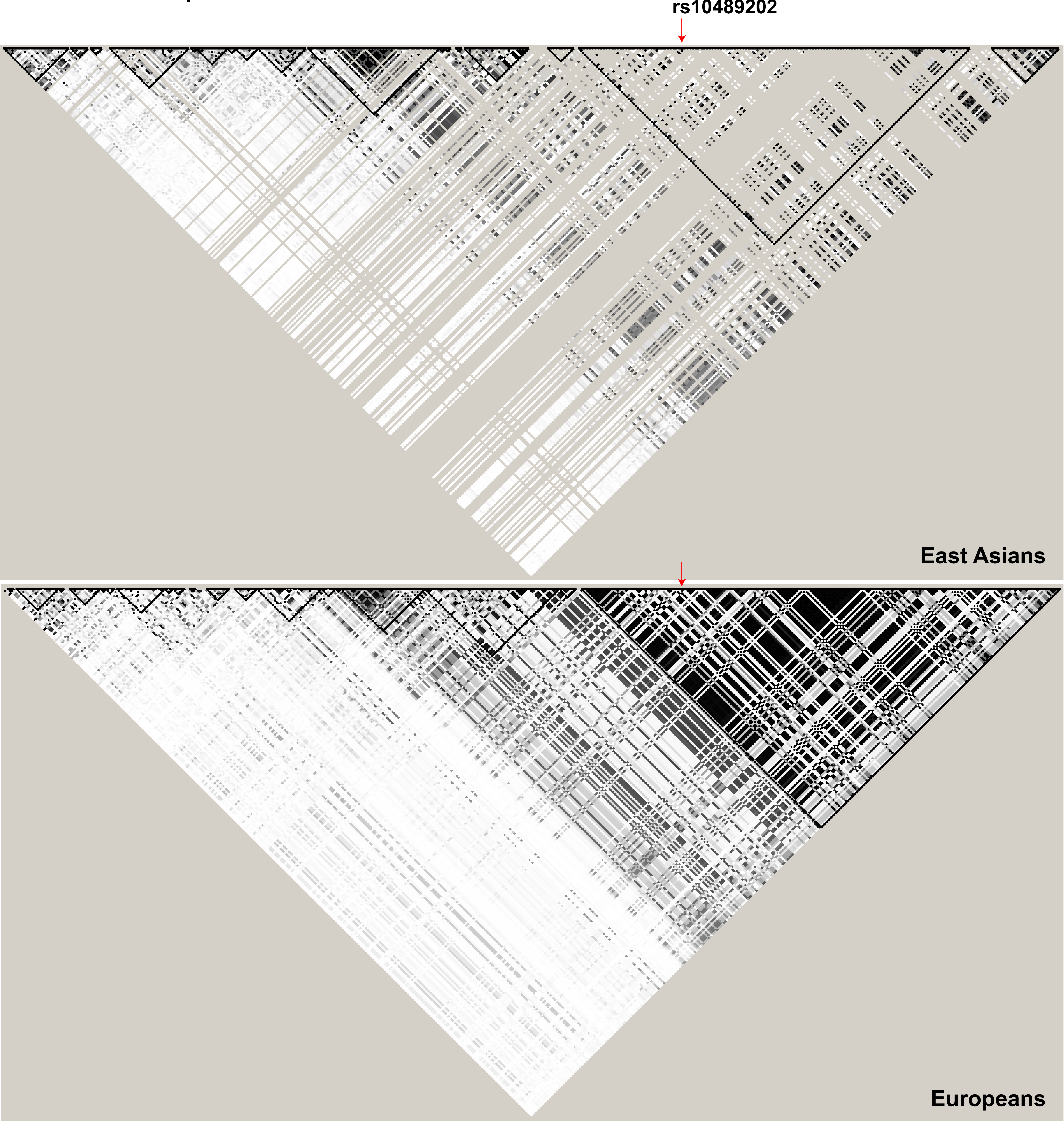

Figure S4. Comparisons of rs10489202 LD SNPs between East Asian and European populations

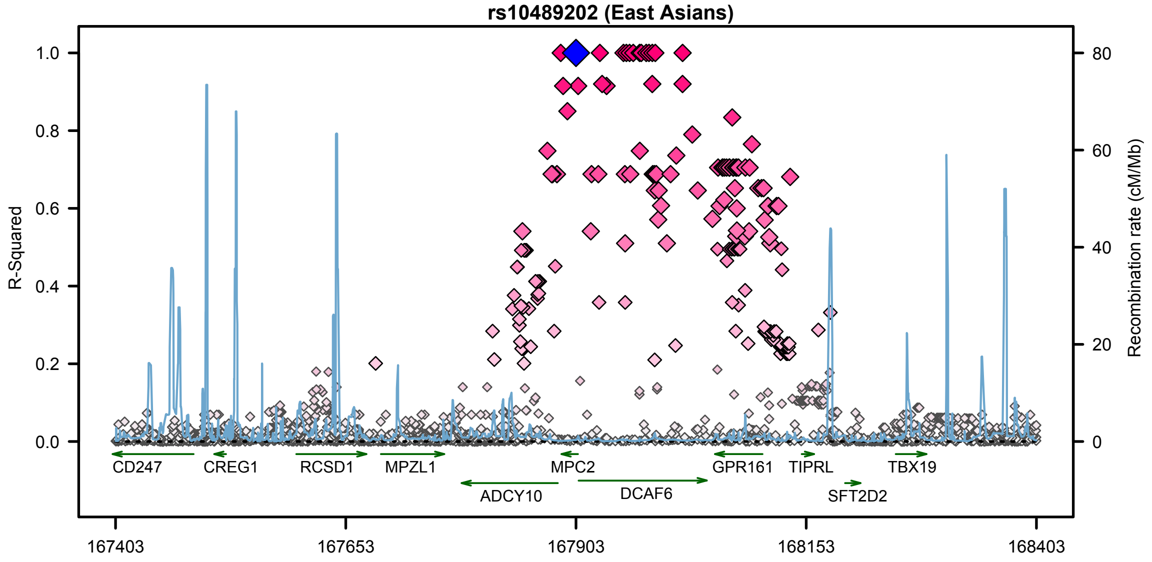

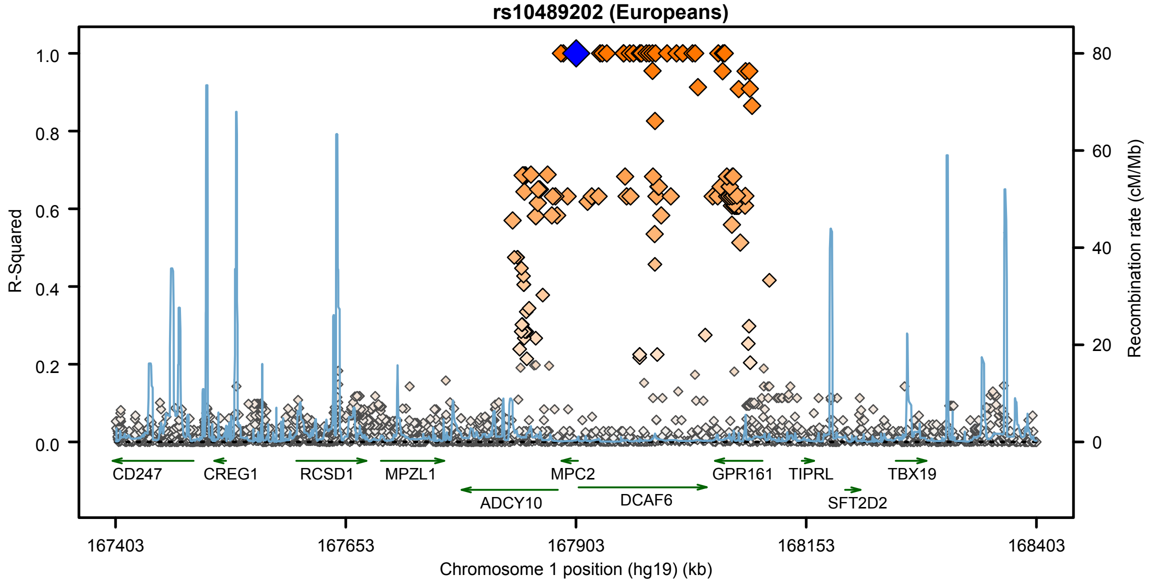

Figure S5. Association results of the SNPs in 1q24.2 with schizophrenia in European populations from PGC2 GWAS

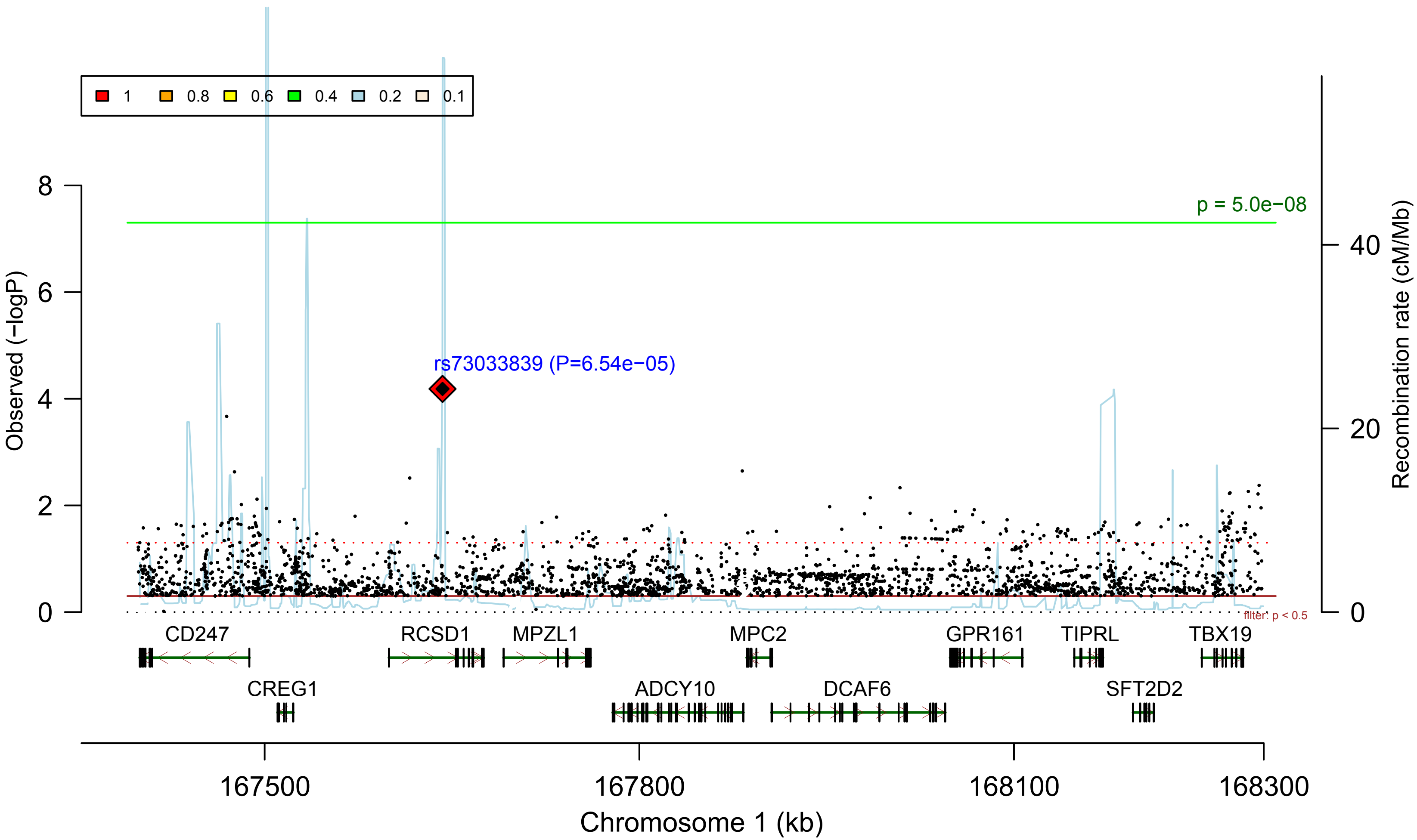

Figure S6. Plot of chromosome region showing a genomic area of LD with rs10489202 in East Asian populations

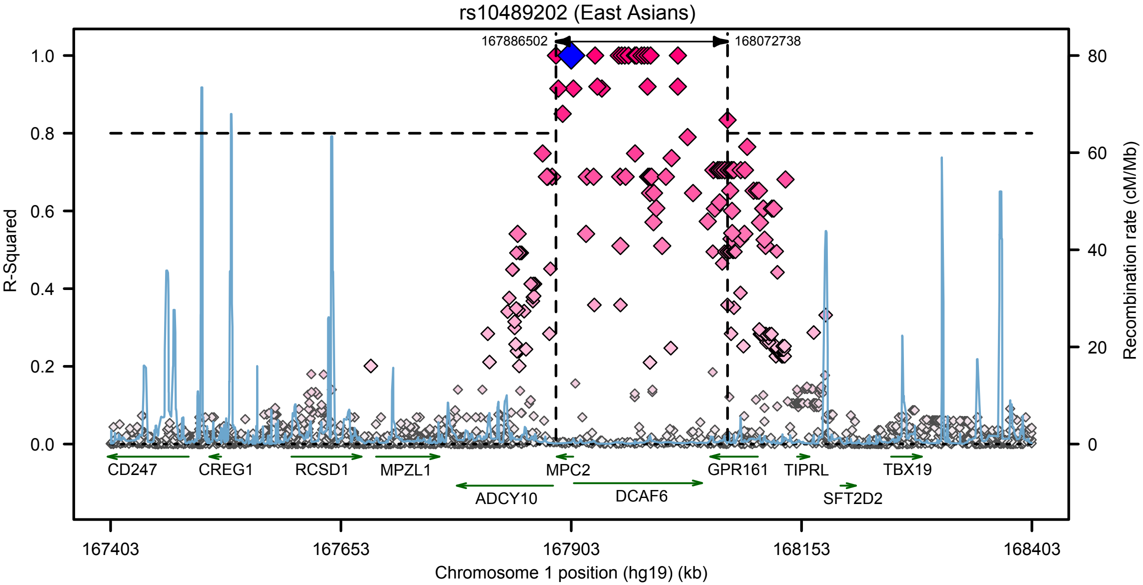

Supplement: Supplementary file 2 — Supplementary Figures [file 41398_2018_211_MOESM2_ESM.pdf]
